# Embryological development of the human cranio-facial arterial system: a pictorial review

**DOI:** 10.1007/s00276-021-02684-y

**Published:** 2021-01-25

**Authors:** Lorenzo Bertulli, Thomas Robert

**Affiliations:** Department of Neurosurgery, Neurocenter of the Southern Switzerland, Via Tesserete 46, 6903 Lugano, TI Switzerland

**Keywords:** Cranial arteries, Embryologic development, Pathophysiology, Variants

## Abstract

The embryological development of the cerebral vasculature is very complex. Historical and also more recent studies based on human embryos, comparative anatomy and cerebral angiographies allowed us to better understand this vasculature development. The knowledge and understanding of such embryological development are important for physicians interested in neurovascular pathologies. Indeed, all vascular variants and almost all vascular pathologies, such as aneurysms, dolichoectasia, atherosclerosis, and neurovascular conflicts could be explained by an alteration during the embryological life. There are also many variants of these vascular structures present in normal developed adults, which are variably associated with pathological entities. Understanding the process which leads to the development of the normal cerebral arterial system in humans is, therefore, very important to have a better knowledge of the possible clinical and surgical implications of these anomalies. In this paper, we review the embryological development of the cranio-facial arterial vasculature from its beginning at approximately days 21–50 of intrauterine life, with pictures illustrating each developmental phase.

## Introduction

In this article, after some definitions and a historical overview, we will describe the general principles of the vascular embryology of the whole cranio-facial arterial system in human. Each paragraph describes one of the seven stages of Padget [27, 28], to make the reading easier. For each stage, general developmental changes, evolution of the anterior circulation and, in the end, the evolution of the posterior and extracranial circulation will be successively addressed. Each stage is summarized by a picture with all the main vessels showing their relationship with each other and with the developing neural and somatic structures.

### Terminology

- Days: the embryo implants in the uterus to start its intrauterine life around 1 week after fertilization. Normally, the embryonic life is divided in weeks: the gestational age (or clinical age) is calculated from the last menstrual period; the post-conception age, on the other hand, refers to days after fertilization. In our work, we will refer to “days” as days after fertilization.
- Stages: with the word “stages” we will only refer to the 7 stages in which Padget arbitrarily divided the arterial system development [27, 28]. They do not correspond to the “Carnegie stages”, which are a standardized system of 23 stages used to provide a unified developmental chronology of the vertebrate embryo (proposed by Streeter in 1942 [35–39] and revised by O’Rahilly and Müller in 1987 [24]). These stages are not based on the size or the number of days of development, but on the developmental changes that occur during the first period of intrauterine life. For the sake of completeness, we specified the corresponding Carnegie stage(s) to the Padget stage in each paragraph.
- Millimeters: the size of the embryos during the various phases is expressed in millimeters of length from the rostral to the caudal point.

Note: in her work, Padget refers to the two main primitive components of the ophthalmic artery as “ventral” and “dorsal”, where ventral is anterior and dorsal posterior [27]. However, if we analyze the true disposition of the vessels, that might be confusing. In fact, the so-called “primitive dorsal ophthalmic artery” comes off more proximally from the primitive internal carotid artery (and, consequently, in a ventral position with respect of the developing head); the so-called “primitive ventral ophthalmic artery” comes off more distally (and so in a dorsal position). In our work, we will maintain the original terminology from Padget, specifying the correct position of the arterial branches when necessary.

### History

Historically, five great authors are known for their thorough studies concerning the cerebral arteries development.

Three of them based their results on the embryos collection of the Carnegie University.

G.L. Streeter in 1921 described the principles leading to cerebral vascular system formation, dividing it in 5 phases: (1) angioblastic stage, (2) arterial, venous and capillary development, (3) penetration of perforators, (4) the construction of more large vessels from a “web” and (5) the histological differentiation [34].

E.D. Congdon in 1922 defined the complete embryology of the aortic arches [5].

D.H. Padget continued the descriptive work of Streeter working on a series of embryos at the Carnegie University, publishing in 1948 a deep and complete study about the embryology of the whole cerebral arterial system starting from the appearance of the internal carotid artery (about 3.5 weeks, 3 mm) to the final configuration (about 7 weeks, 40 mm) [17, 27].

F. Altmann, an Austrian ENT emigrated to the USA in the late 30s, gave his contribution to the field with several works on arterial variants and comparative anatomy [1], and P. Lasjaunias from France is one of the most influential and important names in the field of neurovascular anatomy of the past century, with his immense work published as articles and books on cerebrovascular anatomy, embryology and comparative anatomy [4, 18, 19]. He started from the rigorous study of his large number of angiographies, comparing them to draw up several embryological hypotheses to explain physiological development, anatomical variants and pathological entities.

Embryological stages described by Padget in 1948 [27] are considered as a reference and we chose to follow them to present the key embryological steps of cranio-facial arterial development. With the aim to well understand the embryological development, in the first part of this article, we will describe the development of the embryo’s cranial extremity arteries before the first stage of Padget (1.5–2 mm stage, ≃ 21 days).

### Development of the cranio-facial arterial system

The cerebral vasculature begins to develop quite early in the embryo, during the third week of intrauterine life (21 days ≃ 2 mm), slightly before the heart starts beating [23, 29]. At this stage, the neural tube is not closed yet. The prosencephalon is irrigated initially by the two dorsal aortas (DA), whose rostral part will become the internal carotid arteries (ICA); the mesencephalon and rhombencephalon vasculature develop after, as the vertebro-basilar system appears later [1, 20, 27].

The vascular development consists of 2 main stages: vasculogenesis and angiogenesis [21, 23]. Vasculogenesis is the process by which hemangioblasts are differentiated into angioblasts and the formation of a primitive vascular network; angiogenesis refers to the formation of new capillary vessels from pre-existing blood vessels [23, 29, 41]. The main mechanism of angiogenesis is sprouting, which is mainly driven by hypoxia/ischemia mechanism and related growth factors from the target tissue [3, 23, 29, 31]. As more capillaries are formed, the impedance to flow is reduced in larger arteries; this way, flow-induced remodeling of the vessels supplying a specific parenchymal area is promoted [7, 23].

The development of the circulatory system supplying blood to the brain begins with the formation of the 6 pairs of primitive branchial arch arteries, subsequently undergoing heavy modifications during development [1, 23].

### The arterial arch and proximal arterial branch development

**Fig. 1:**
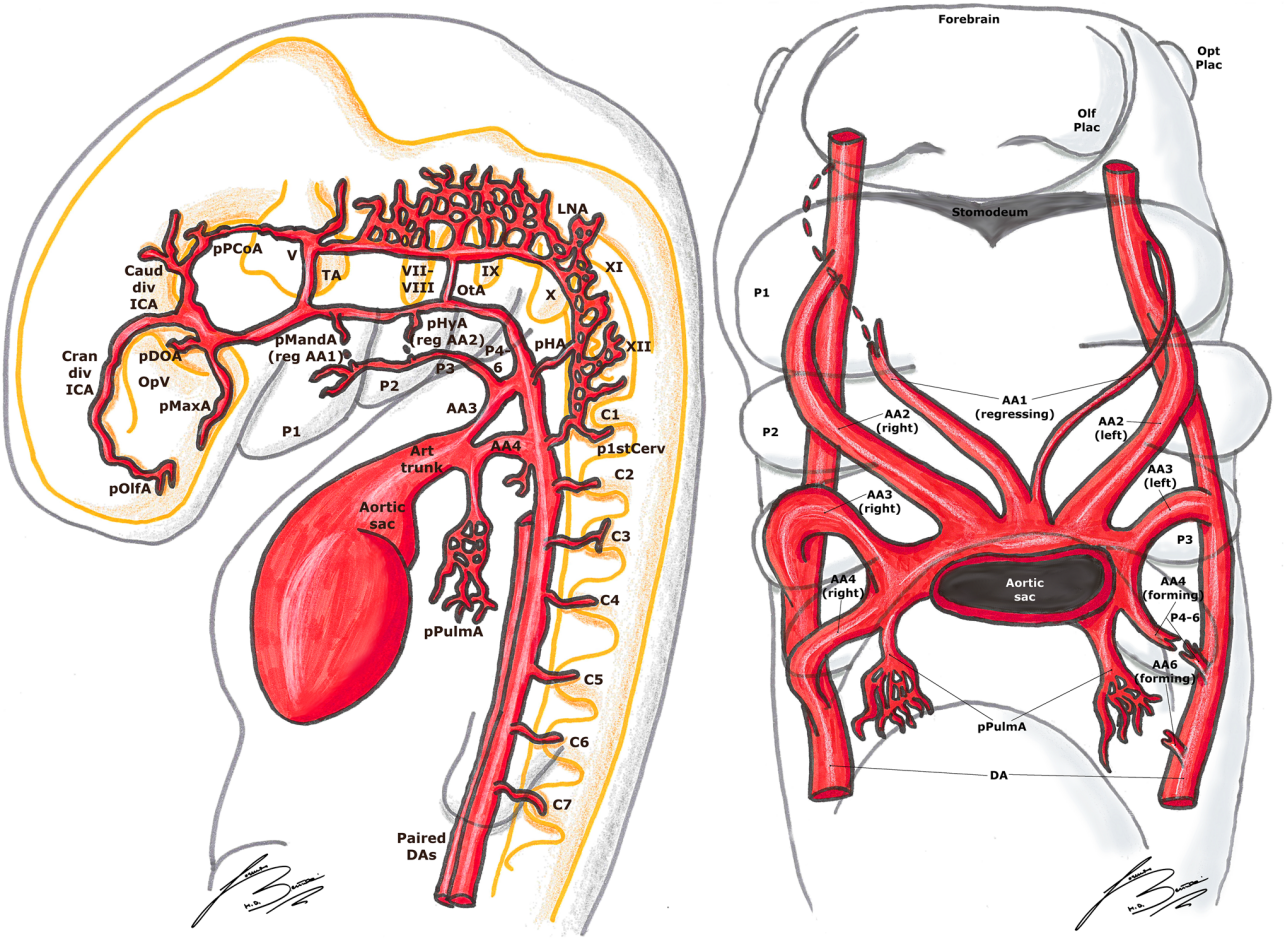
Padget stage 1; lateral (left) and frontal (right) view of a 4–5 mm,  ≃  30-day-old embryo. In gray, the outer structure of the embryo is outlined; in yellow, the developing nervous system is represented. Developing cranial nerves are numbered from V to XII, developing spinal roots are listed from C1 to C7 and pharyngeal arches from P1 to P4-6.

**Fig. 2:**
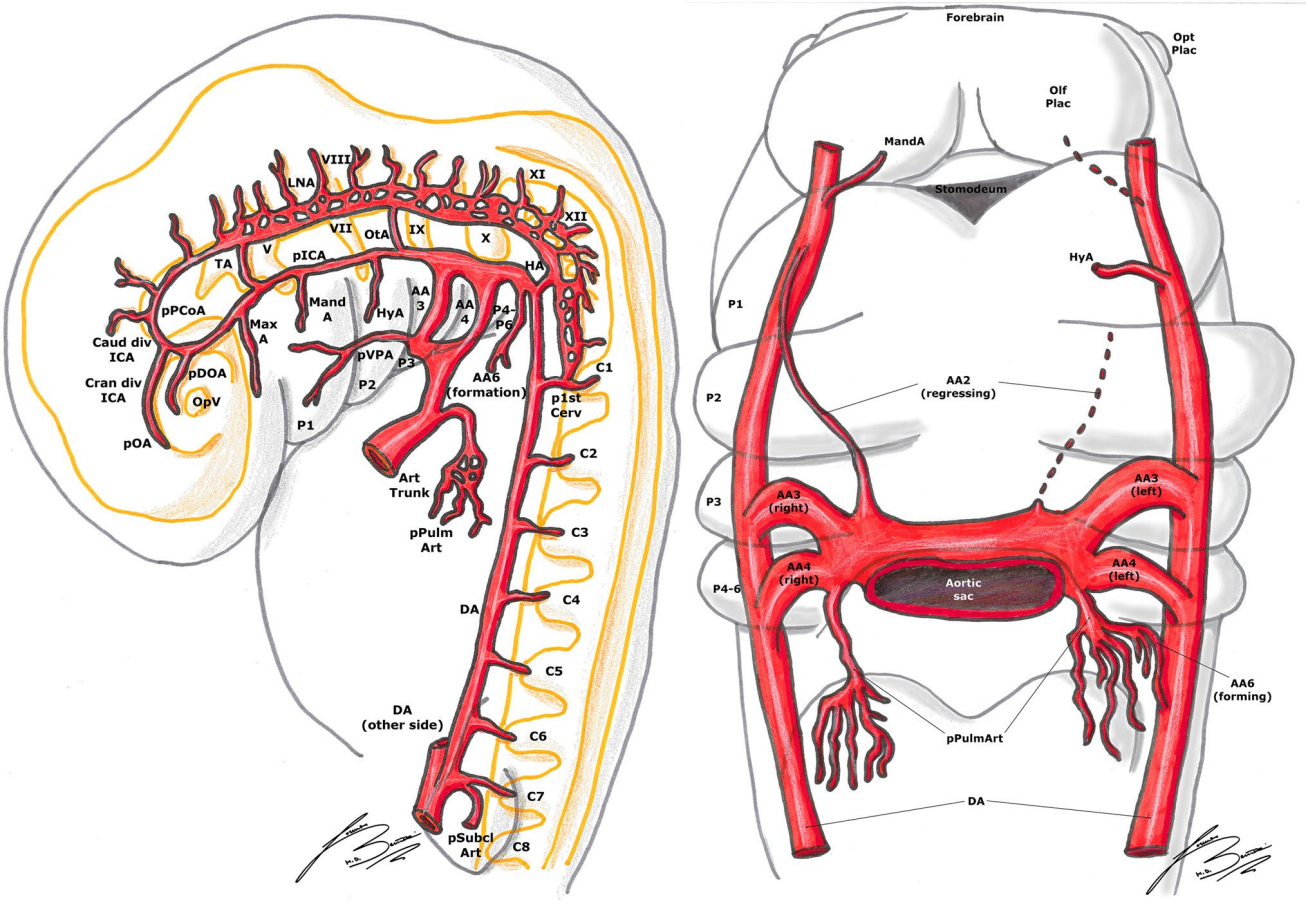
Padget stage 2; lateral (left) and frontal (right) view of a 5–6 mm, ≃ 31-day-old embryo. In gray, the outer structure of the embryo is outlined; in yellow, the developing nervous system is represented. In gray, the outer structure of the embryo is outlined; in yellow, the developing nervous system is represented. Developing cranial nerves are numbered from V to XII, developing spinal roots are listed from C1 to C8 and pharyngeal arches from P1 to P4-6.

**Fig. 3:**
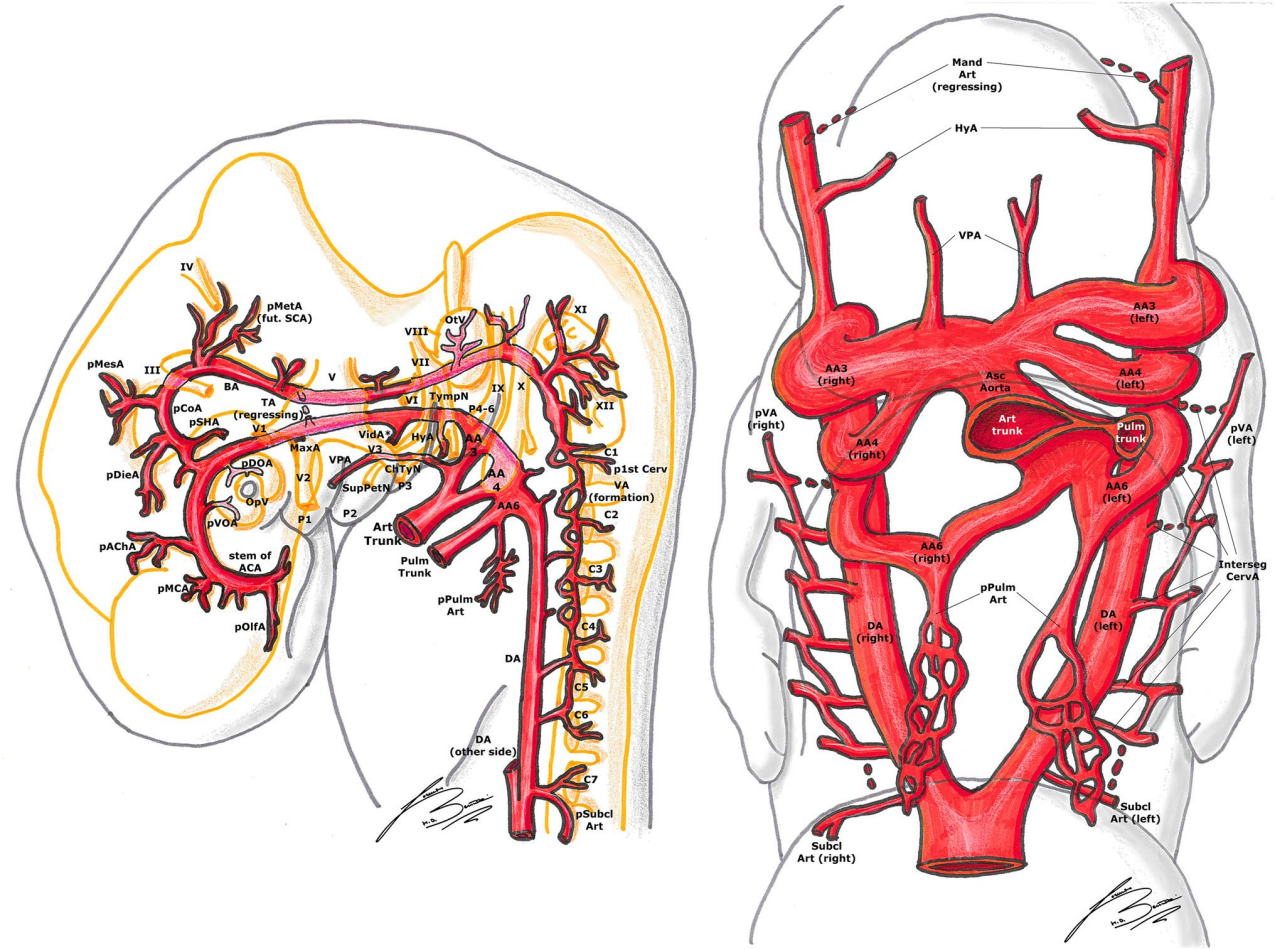
Padget stage 3; lateral (left) and frontal (right) view of a 7–12 mm, ≃ 33-day-old embryo. In gray, the outer structure of the embryo is outlined; in yellow, the developing nervous system is represented. In gray, the outer structure of the embryo is outlined; in yellow, the developing nervous system is represented. Developing cranial nerves are numbered from III to XII, developing spinal roots are listed from C1 to C7 and pharyngeal arches from P1 to P4-6.

The human embryo has six pair of visceral arterial arches (AA) [1, 5, 14, 23, 25, 27, 33]. Each AA arises ventrally from the bulbus arteriosus (aortic sac) and courses in the corresponding visceral branch, to end dorsally in the DA [1, 5, 23, 27, 33]. They are present until about the 13 mm stage; the development starts from front to back, but they are never all present at the same time [1, 14, 27, 33]. For example, the first AA regresses during the completion of the fourth, and the second disappears before the sixth is completely closed [5, 27] (Figs. 1,2, 3).

Controversies exist about the existence of a fifth arterial arch: only the first four pharyngeal arches, grooves and pouches are distinct structures. Nevertheless, arch arteries develop caudal to the fourth arch. The pulmonary arch caudal to the fourth arch artery is named a “sixth arch artery” because of its phylogeny, even when a fifth arch artery is not present [5]. Some recent works have tried to explain several anomalies, such as double-barrel aorta, with the presence of a persistent fifth arch artery; despite in the most cases other mechanisms seem to explain these anomalies, in rare cases “true” fifth arterial arches have been described [2, 12, 13].

**Fig. 4:**
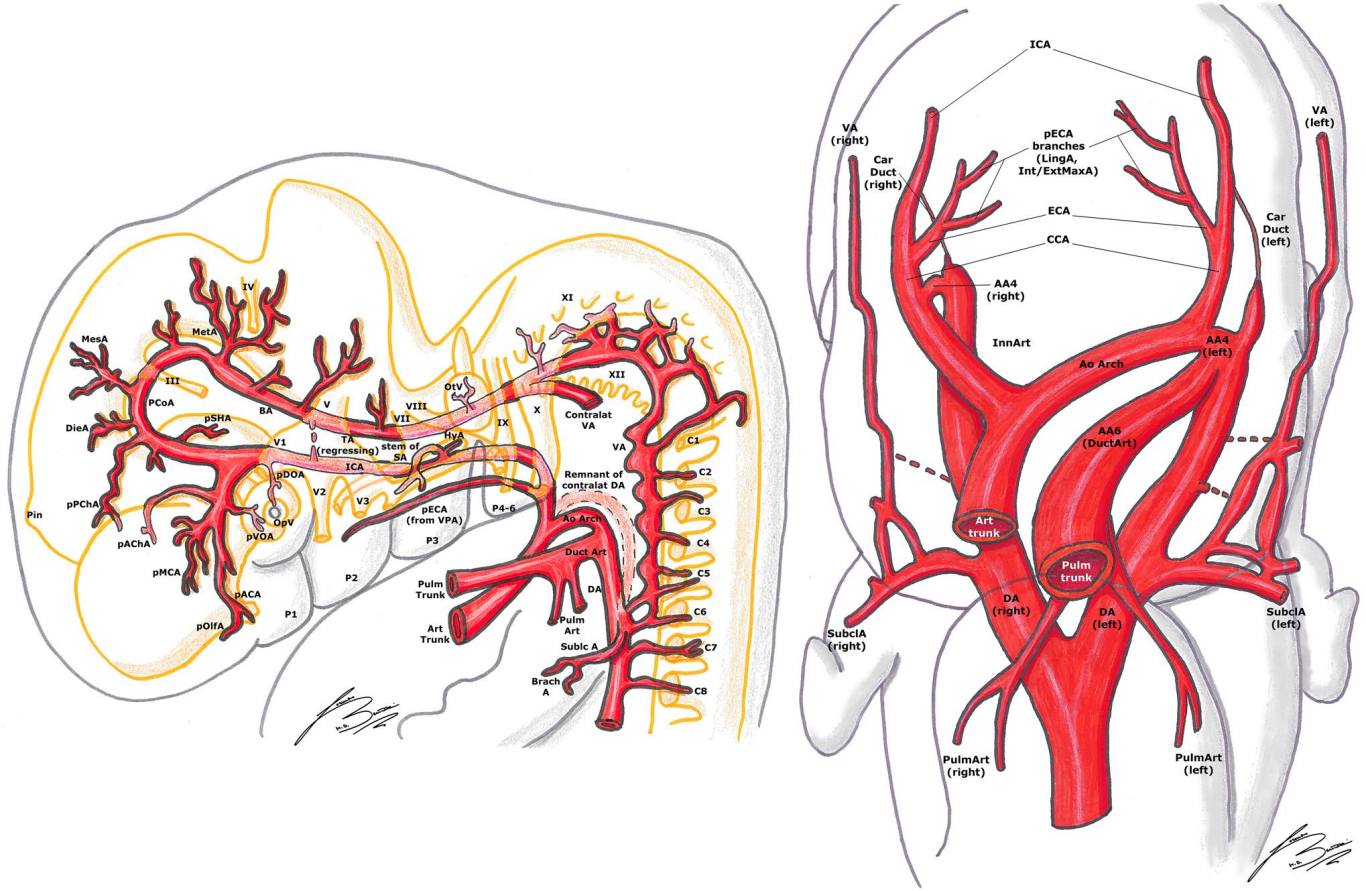
Padget stage 4; lateral (left) and frontal (right) view of a 12–14 mm, ≃ 36-day-old embryo. In gray, the outer structure of the embryo is outlined; in yellow, the developing nervous system is represented. In gray, the outer structure of the embryo is outlined; in yellow, the developing nervous system is represented. Developing cranial nerves are numbered from III to XII, developing spinal roots are listed from C1 to C8 and pharyngeal arches from P1 to P4-6.

**Fig. 5:**
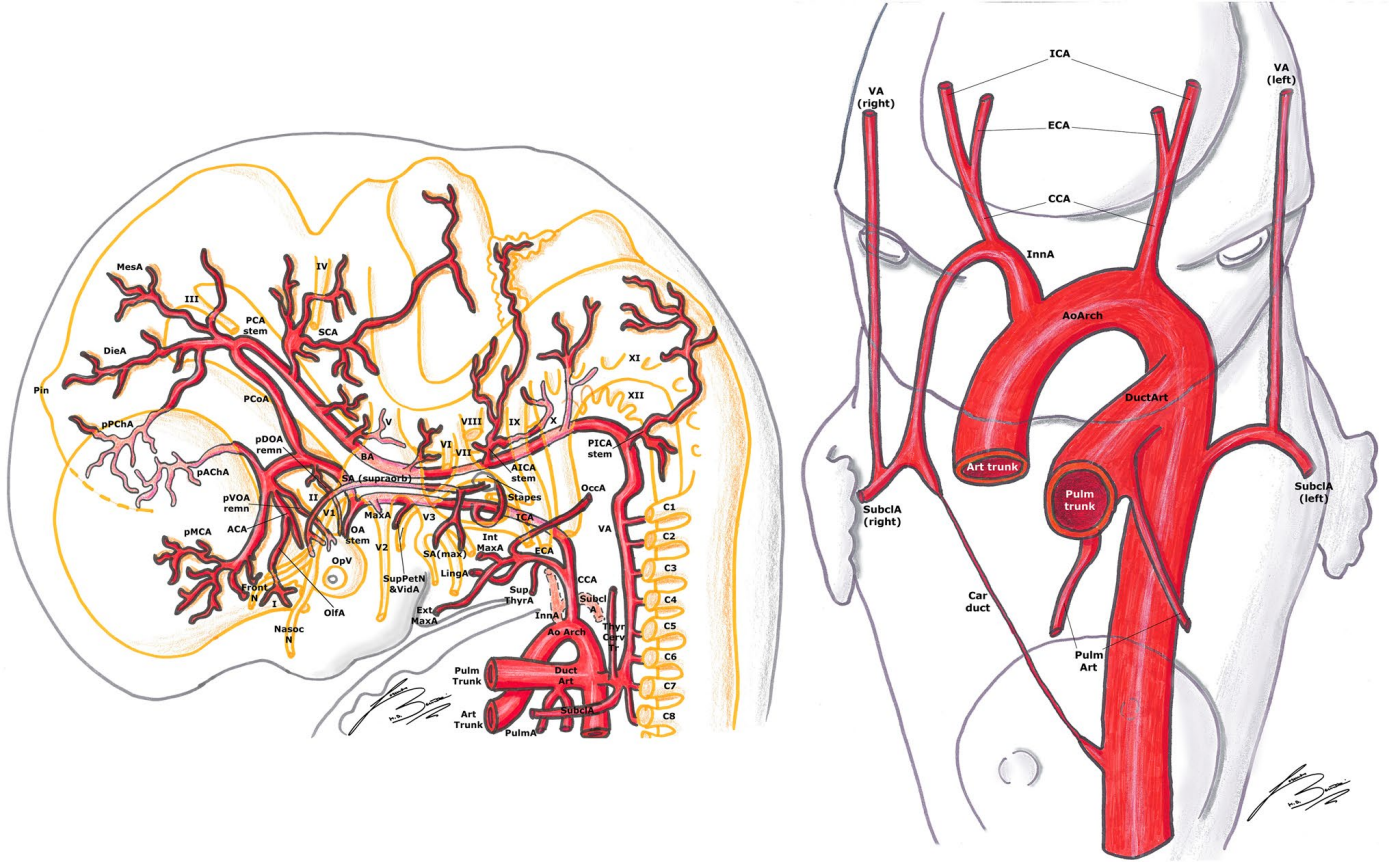
Padget stage 5; lateral (left) and frontal (right) view of a 16–18 mm, ≃ 40-day-old embryo. In gray, the outer structure of the embryo is outlined; in yellow, the developing nervous system is represented. In gray, the outer structure of the embryo is outlined; in yellow, the developing nervous system is represented. Developing cranial nerves are numbered from I to XII, developing spinal roots are listed from C1 to C8. Pharyngeal arches are becoming the respective fetal derivatives.

In embryos longer than 13 mm, the portion of DA between the third and fourth AA disappears on both sides of the embryo [1, 27]. The same does the segment between the fourth AA and the junction with left DA, but only on the right side [1, 27]. This way, the left horn of the aortic sac and the fourth AA form definitive aortic arch (AoArch), and the right horn of the aortic sac becomes the arteria anonyma (future brachio-cephalic trunk). The third AA will become the ICA [1, 23, 27]. Its main trunk is formed by the segment of the DA between the first and third AA. The external carotid arteries (ECA) develops later from the ventral pharyngeal arteries [1, 20, 23, 27] (Figs. 4, 5).

### The development of the cranio-facial arteries (stage-by-stage development)

#### “Pre-Padget” stage ( ≃ 21 to ≃ 28 days, from 1.5–2 to 4 mm); Carnegie stages 10–12

At the beginning of the “pre-Padget” stage, after about 21 days of intrauterine life, both the ventral aortas (from the aortic bulb) and DAs are connected by the first AA [1, 20, 27]. In the next days, the second AA develops and the first two AAs are thus well visible [5, 27]. The two DAs course along the whole embryo, giving segmental branches to each somite [5, 27]. The primitive heart starts beating after about 23 days [5, 20, 27].

At about 4 mm length, ≃ 28 days of intrauterine life, the two ventral aortas (or the two horns of the aortic bulb) are still present; the DAs are beginning to fuse, starting from their caudal end in a “zipper-like” fashion to form the definitive aorta [20, 27]. The first four AAs are visible; the first one is beginning to disappear while the fourth is being completed [1, 27].

#### 4–5 mm stage (≃ 30 days), Padget stage 1; Carnegie stage 13

At this stage, the optic vesicle (OpV, whose formation began around the 18th day) is well visible. The first AA is completely regressed, and the regression of the second has begun; they will become the mandibular (MandA) and hyoid arteries (HyA), respectively [14, 20, 25–27, 33] (Fig. 1).

As mentioned before, the primitive ICA is beginning its development from the DA located between the first three AAs and from the third AA itself [14, 20, 23, 25, 27]. At the level of the trigeminal ganglion, there is a first division into two branches: (1) the primitive trigeminal artery (TA), which extends dorsally to join the longitudinal neural artery (LNA) on the hindbrain wall; (2) the continuation of the carotid, extending cranially and medially towards Rathke’s pouch [14, 20, 23, 25, 27, 30, 33, 40]. This branch joins its counterpart on the opposite side, to which is connected by a plexus [20, 27, 30]. The primitive maxillary artery (MaxA) is also developing at the base of Rathke’s pouch, with a ventral course along the forebrain wall towards the OpV [11, 27]. Near to the most cranial part of the OpV, the ICA bifurcates in two branches: the caudal branch will become the posterior communicating artery (PCoA) and, in the future stages, the posterior cerebral artery (PCA); the cranial branch (future anterior cerebral artery, ACA) curves around the frontal part of the vesicle to terminate in the olfactory area [20, 23, 27, 30] (Figs. 1, 2).

The primitive ophthalmic artery (OA) appears like a small branch sprouting near the carotid division at the medial side of the optic vesicle, but the supply to the vesicle itself is highly plexiform in this stage and contributed by the carotid and the primitive MaxA [11, 20, 27, 30] (Figs. 1, 2).

In the posterior cerebral region (hindbrain), the two LNAs appear, with a strip of non-vascular tissue in the midline [10, 14, 20, 23, 27, 30]. They are supplied cranially by the TA and otic artery (OtA) and caudally by the hypoglossal artery (HA) and first cervical segmental artery (p1stCerv, also called proatlantal artery) [10, 14, 20, 23, 27, 30] (Figs. 1, 2).

#### 5–6 mm stage (≃ 31 days), Padget stage 2; Carnegie stage 14

The ICA, extending from the third AA, is now well delineated. The MandA (remnant of the first aortic arch) is now regressing [14, 20, 25–27, 33]. The HyA (remnant of the second aortic arch) is quite thick, and will become the stem of the stapedial artery (SA) in the future stages [14, 20, 25, 27, 33]. In the median part of the embryo, between the first two AAs, there is a big vessel, called the ventral pharyngeal artery (VPA). This vessel extends from the aortic bulb to a cranial and lateral direction towards the mandibular root of the fifth nerve [27]. Once the AAs regress, this branch becomes the primitive ECA [20, 23, 25–27, 33]. The primitive MaxA follows the lateral margin of Rathke’s pouch to reach the ventromesial aspect of the tip of the prosencephalon, terminating in a plexus [11, 27] (Fig. 2).

Lateral to the origin of the primitive ventral OA (pVOA, the more distal branch), more proximal to the primitive carotid bifurcation, a longer branch develops passing over the dorso-caudal margin of the optic disc to the primitive lens. This is the so-called primitive dorsal OA (pDOA, the more proximal branch) [11, 20, 27, 32]. Consequently, at this stage, the optic supply is dual (pVOA plus pDOA) [11, 27, 32] (Fig. 2).

The caudal division of the ICA has formed a second anastomosis with the cranial end of the LNA at the mesencephalon, forming the definitive PCoA [14, 20, 23, 25, 27, 30] (Fig. 2).

Consequently, the TA begins to involve, being replaced by the PCoA, and the two LNAs begin to fuse to form the primitive basilar artery (BA). The hypoglossal arteries (HA) also begin their regression; this is because the most part of the blood supply to the caudal LNAs and BA comes from the first cervical segmental branches. A temporary vessel supplied cranially by the lateral branch of the BA (i.e., TA) and caudally by the first cervical segment artery often becomes an accessory anastomosis between BA and vertebral arteries (VAs) [10, 14, 20, 25, 27] (Fig. 2).

#### 7–12 mm stage ( ≃ 33 days), Padget stage 3; Carnegie stages 15–16

The ICA is quite large in the cerebral part, while is thinner at the level of the third aortic arch. The HyA courses caudally, laterally and ventrally to reach the area between the seventh nerve root and the glossopharyngeal ganglion (between the second and third branchial arches). The MandA is now regressed almost completely. The VPA supplies the first two pharyngeal arches [20, 25–27] (Fig. 3).

The pDOA is now fully developed, supplying a plexus to the OpV; the shorter pVOA is also well recognizable, rising near the junction between the ICA and PCoA [11, 20, 27, 32]. Several branches now appear from the cranial division of the ICA: the most important of these is the primitive anterior choroidal artery (AChA), coursing along the outlining choroid fissure in the diencephalon [20, 27, 30, 43]. Some small sprigs arise distally to this branch representing the stem of the MCA [20, 27]. More distally, just before the termination of the ICA at the olfactory sac, the primitive anterior cerebral artery (ACA) is beginning its development [14, 20, 25, 27, 30] (Fig. 3).

From the caudal end of the primitive PCoA, two branches emerge right next to the third nerve: one supplies the diencephalon (diencephalic artery, DieA) and gives a posterior choroidal branch (PChA) which is directed towards the AChA, while the other one supplies the mesencephalon (mesencephalic artery, MesA) [20, 27] (Figs. 3, 4).

**Fig. 6:**
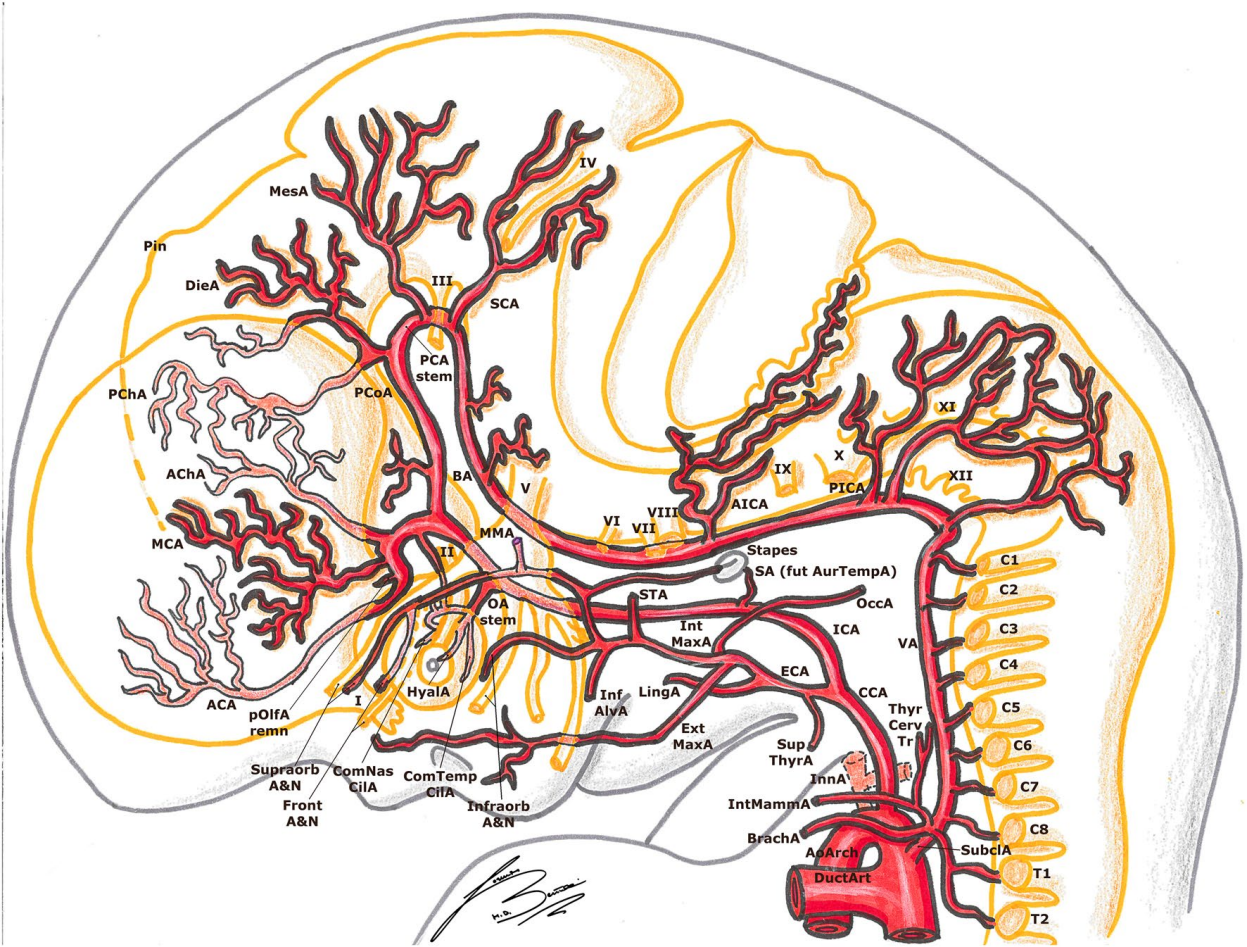
Padget stage 6; lateral view of a 20–24 mm, ≃ 45-day-old embryo. In gray, the outer structure of the embryo is outlined; in yellow, the developing nervous system is represented. In gray, the outer structure of the embryo is outlined; in yellow, the developing nervous system is represented. Developing cranial nerves are numbered from I to XII, developing spinal roots are listed from C1 to T2. The arterial “ring” or loop around the optic nerve is now visible.

The PChA from the PCoA anastomoses with the AChA and the choroidal branches of the ACA in the choroidal plexus at level of the interventricular foramen. Later on, the ACA pericallosal branches to the choroidal plexus elongate and regress. This AChA–PChA anastomosis is named “limbic arterial arch” and in some cases may persist in the adult life [9, 27, 43] (Figs. 3, 4, 5, 6).

At this stage, the VA begins its formation by a transverse anastomosis between the six upper cervical segmental arteries; meanwhile, the aortic end of each cervical segmental branch is progressively occluding and each segmental branch regresses [14, 20, 23, 25, 27, 30]. It is notable that each transverse anastomosis courses from the more proximal end of one segmental branch to the more distal end of the next branch (in a cranial direction) (Figs. 3, 4).

From the cranial end of the BA, which is still quite irregular in shape and formed by some “islands”, rises the metenchephalic artery (MetA) or primitive superior cerebellar artery (SCA), supplying the region of the fourth nerve and metencephalon. Some symmetrical branches leaving from the BA supply the developing cranial nerves [20, 27] (Figs. 3, 4).

#### 12–14 mm stage (≃ 36 days), Padget stage 4; Carnegie stage 17

This stage is important from a developmental point of view for the higher vertebrates. The definitive conformation of the cranial division of the ICA is well identifiable. The first collateral of this cranial division is the AChA [20, 27, 30, 43]. The second one is the primitive MCA, and the next and more distal branch is the stem of the ACA, at the terminal end of the cranial ICA division (the primitive olfactory artery, pOlfA) [11, 20, 25, 27]. This artery divides into a branch to the nasal fossa, and another and more medial one to the emerging olfactory nerve root; this second branch is the future definitive ACA, and in some embryos is joined with its contralateral homolog by plexiform anastomoses in the midline, the “sketch” of the anterior communicating artery (ACoA) [11, 14, 20, 25, 27, 30]. It is notable that, in this phase, the terminal branch of the ICA appears to be the ACA, whereas in the adult configuration, the MCA is the most important branch of this artery and the ACA seems to be a “collateral” (Figs. 3, 4).

The pDOA is now longer and gives two branches: the common temporal ciliary artery (ComTempCilA, Fig. 6), to the caudo-dorsal aspect of the optic cup, and the hyaloid artery (HyalA, future central retinal artery), which enters the ocular cleft. The pVOA is also elongated and its distal part will form the common nasal ciliary artery (ComNasCilA, Fig. 6). The definitive stem of the OA has not yet begun its development [11, 20, 27, 32].

This stage is characterized by the formation of the SA. The formation of this branch starts from the HyA, which gives a collateral branch passing through the primitive stapes, between the first pharyngeal pouch to the seventh nerve, to descend in the mandibular substance [20, 27, 33] (Fig. 4).

The thyroid (ThyrA) and lingual (LingA) arteries, branches of the ECA, arise from the VPA and begin their development in most of the embryos at this stage (Fig. 5). The obliteration of the two ventral aortas between the third and fourth AA is almost complete, giving rise to the common carotid artery (CCA) [20, 26, 27] (Figs. 4, 5).

#### 16–18 mm stage (≃ 40 days), Padget stage 5; Carnegie stages 18–19

At this stage, we can see the ongoing clarification of the adult vascular configuration. The descent of the heart and the large proximal arterial trunks into the thorax is now almost completed (Figs. 5, 6).

The CCA is nearly fully elongated, the VA is more regular and straightened and its origin from the subclavian artery (SubclA), shifting cranially and opposite to the pulmonary arch (the sixth AA), is defined (Fig. 5).

The pOlfA (the lateral terminal part of the anterior division of the ICA, as seen in the previous stage) is quite large and gives off a branch for the olfactory nerve, but now its medial counterpart, the stem of the definitive ACA, is clearly bigger [27] (Figs. 5, 6).

The MCA is now a quite big trunk with branches spreading out to the growing hemisphere. The AChA and PChA terminate in the choroidal fissure at the diencephalic roof [9, 27, 43] (Figs. 5, 6).

The permanent stem of the OA appears; although the exact formation of this artery is still debated and controversial, we here report the most accepted hypothesis. The SA is now entirely developed, giving two mayor branches. The first one is the maxillo-mandibular branch (ventral), from which the MandA and MaxA (or infraorbital) arteries arise. The second one is the supraorbital branch (dorsal), which passes lateral to the trigeminal ganglion to reach the primitive orbit. Here, this branch anastomoses with branches of the pDOA (ComTempCilA and HyalA) and of the pVOA (ComNasCilA). Therefore, the definitive OA consists of two different parts of the ICA: (1) the supraorbital branch of the SA, which gives the orbital arteries and (2) pDOA and pVOA, which give the ocular branches. As these anastomoses form, the original stem of the pDOA regresses (becoming part of the inferolateral trunk) and the artery itself makes a sort of caudal “migration” along the ICA. As a result, the definitive stem of the OA develops in its final position [11, 20, 27, 32, 33] (Figs. 5, 6).

It is usually found a dorsal remnant of the MandA which runs along the future greater petrosal nerve in the pterygoid (or Vidian) canal, to reach the pterygopalatine fossa: these are the adult Vidian artery (VidA) and nerve. There usually is also a primitive maxillary branch, directed medially and cranially towards the caudal border of the hypophysis, which contributes to the inferior hypophyseal artery [20, 25–27] (Figs. 5, 6).

Together with the appearance of the definitive CCA, several ECA branches begin their development: the thyroid (ThyrA), lingual (LingA), occipital (OccA) and external maxillary (ExtMaxA) arteries appear. The internal maxillary artery (IntMaxA) anastomoses with the mandibulo-maxillary branch of the SA [11, 20, 26, 27, 33] (Figs. 5, 6).

The diencephalon is supplied by two large branches: the more anteroventral is the PChA, the other one is the DieA, which courses towards the primitive pineal region. The mesencephalon is also supplied by two branches (MesA and lateral branch of PChA) [27] (Figs. 4–6).

In this stage, the PCA starts to develop as the caudal segment of the PCoA, and can be considered a branch of the PCoA such as the PChA or the hindbrain arteries. During the development of the cerebral hemispheres, the PCA progressively enlarges and the other smaller arteries become PCA branches, as we can see in the adult configuration [14, 20, 25, 27, 30] (Fig. 5).

The BA branches also develop as the mesencephalon and the fourth ventricle region differentiate. The SCA divides in a mesial branch coursing caudal to the future fourth nerve and a lateral one continuing over the cerebellar lip. The stem of the future antero-inferior cerebellar artery (AICA) arises at the level of the eight nerve, ending at the choroid plexus of the fourth ventricle. More caudally, many small branches emerging from the VA with many anastomoses pass through the rootlets of the vagus and accessory nerves. One bigger branch of the VA runs cranially to the choroid plexus along the medulla, constituting the stem of the future postero-inferior cerebellar artery (PICA) [20, 27, 30] (Figs. 5, 6).

#### 20–24 mm stage (≃ 45 days), Padget stage 6; Carnegie stages 20–21

In this stage, as the head begins to lift away from the chest, acquiring more human features, and the cerebral hemispheres expand, the final configuration of the adult circle of Willis is almost perfectly recognizable.

The IntMaxA (a branch of the ECA, as mentioned before) now anastomoses with the lower division of the SA (maxillo-mandibular branch), going lateral to V3 nerve. When the ventral (infraorbital) division of the SA becomes surrounded by the auriculotemporal nerve, the part remaining over the anastomosis with the IntMaxA becomes the stem of the middle meningeal artery (MMA). The dorsal (supraorbital) division of the SA, laterally to the trigeminal ganglion, constitutes the extension of the MMA. At this point, the proximal segment of the SA (medial to the stapes) begins its regression: thus, the maxillo-mandibular branch of the SA (and so the MMA too) becomes an ECA branch [11, 20, 27, 32, 33] (Fig. 6).

At the level of the OA, the primitive ComTempCilA and HyalA anastomose with the ComNasCilA of the primitive ventral OA to constitute a sort of “arterial loop” around the optic nerve. This loop is also connected with the supraorbital branches of the SA (which has become the MMA, as seen above). As a result, the OA gains a more dorsal position to the optic nerve and gets its orbital branches (that come from the SA) [11, 20, 27, 32, 33] (Fig. 6).

**Fig. 7:**
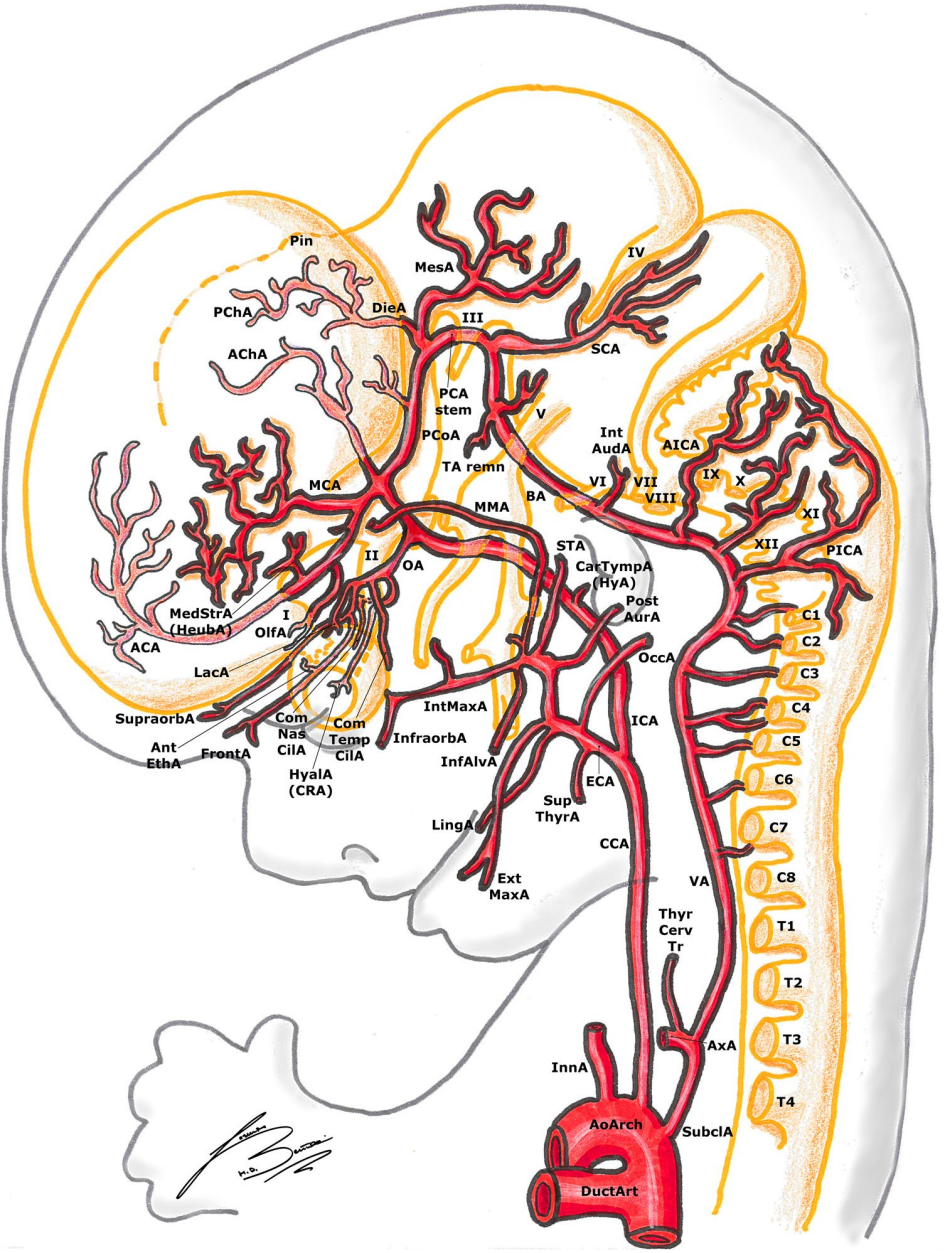
Padget stage 7; lateral view of a 40 mm, ≃ 50-day-old embryo. In gray, the outer structure of the embryo is outlined; in yellow, the developing nervous system is represented. In gray, the outer structure of the embryo is outlined; in yellow, the developing nervous system is represented. Developing cranial nerves are numbered from I to XII, developing spinal roots are listed from C1 to T4. The definitive pre-term vessel configuration is almost completed.

The two ACAs approximate and course upwards between the two expanding cerebral hemispheres. In some embryos, a small branch to the choroid plexus can be found, before the development of the corpus callosum [25, 27]. The persistence of such branch in the human adult is extremely rare, as it regresses while the corpus callosum develops and the artery moves more cranially and dorsally [14, 25, 27]. The ACoA is much less plexiform and almost completed, with a branch to the commissural plate; the course of this branch is more caudal than ACAs, and it can become an unpaired or accessory ACA in the adult [23, 27]. The pOlfA is now a small branch which follows the olfactory nerve in the nasal cavity; a larger branch, arising from the primitive stem of the olfactory artery, has a more lateral course to supply the forming basal ganglia, called the medial striate branch. This is the so-called “recurrent artery” (or Heubner’s artery). This artery is formed by components of the anastomosis between the primitive olfactory artery and its “collateral” ACA branch; it leaves the ACA at the level of the ACoA (or slightly more distally), extends towards the anterior perforated substance and enters it to supply the anteroinferior part of the striatum and internal capsule [11, 20, 22, 27, 42] (Fig. 7).

The PCoA (which initially was a direct continuation of the ICA to the BA) now moves cranially and posteriorly becoming a true “collateral” leaving the ICA with a 90° angle.

The AChA and PChA are connected by several anastomoses at the level of the diencephalon and choroid plexus, as explained before [9, 20, 27, 43]. With the progressive enlargement of the basal ganglia, the contribution of the AChA for the plexus becomes smaller, as this artery’s main supply remain the pallidus, the posterior limb of the internal capsule, the optic tract, the caudate tail and the amygdala, with some cortical branches to the uncinate gyrus (Figs. 6, 7).

The stems of both AICA and PICA are still difficult to recognize among many arterial branches supplying the posterior part of the hindbrain, which still have a quite plexiform configuration. This is because the cerebellar hemispheres have not developed yet. The great number of variants in the vertebro-basilar circulation is explained by the late formation of the cerebellar hemispheres, which leads to this highly plexiform arterial configuration with persistence of remnants of the vertebro-basilar anastomoses described from stage 2 [14, 20, 23, 25, 27, 30].

#### 40 mm stage (≃ 50 days), Padget stage 7; Carnegie stages 22–23

In this stage, in almost all the embryos, the adult stem of nearly all the main arterial branches can be identified. The circle of Willis is now fully recognizable also from the basal view, thanks to the definitive position of the PCoA. As mentioned before, the length, size and direction of the posterior branches (PCA, AICA, PICA) are determined by the development of the cerebellum [27, 30].

As seen before, we can find an accessory branch of the ACA close to the midline (the former “median artery of the corpus callosum”) [23, 27].

In this phase, the OA takes the blood supply of the orbit as the SA stem has disappeared during the previous stage. The lacrimal artery (LacA) is the last appearing orbital branch of the OA, originating from the orbital segment of the supraorbital division of the SA. The extraorbital part of the supraorbital SA division becomes a collateral of the anterior branch of the MMA (which is now an ECA branch, as seen before). In the adult, this is a site of frequent anastomoses between ICA (LacA) and ECA (anterior branch of the MMA). In the latter stage, the HyalA becomes the central artery of the retina and takes charge of the blood supply to the deep ocular structures [11, 20, 27, 32, 33] (Fig. 7).

The PCoA is quite large in this stage, and in this phase, the adult configuration known as “fetal PCoA” can take shape: the PCoA can remain bigger (as the original caudal end of the posterior division of the ICA) and give off the homolateral PCA, with a very small branch connecting the divisional branch of the basilar (the first segment of the PCA in the normal configuration) [20, 23, 25, 27].

### Beginning of the fetal stage

At 7–8 weeks of gestational age, the embryological phase is over and we begin to speak about “fetus” [8, 24].

The cerebrum is now a “privileged” organ, being composed by an almost complete arterial system and receiving a more oxygenated blood compared to the other organs.

In fact, well-oxygenated blood coming from the placenta passes from the umbilical vein through the *ductus venosus* (or left half of the liver), bypassing the inferior vena cava to reach directly the left atrium and ventricle through the foramen ovale, and then goes up the ascending aorta to the ICAs. Deoxygenated blood from the superior and inferior vena cava, forming the so-called “via dextra” through the right atrium and ventricle, passes in the pulmonary trunk and *ductus arteriosus* (DuctArt), to mix with the rest of the oxygenated blood coming from the ascending aorta, to supply the other organs and limbs [6, 15, 16, 27].

## Conclusion

In this paper, we tried to sum up the main stages and concepts of the embryological development of the blood supply to the brain, to have a better understanding of the normal anatomy and, doing so, make it easier to recognize and understand some of the most important anatomical variants or pathologies.

## Abbreviations

AA: Arterial arch
ACA: Anterior cerebral artery
AChA: Anterior choroidal artery
AICA: Antero-inferior cerebellar artery
AntEthA: Anterior ethmoidal artery
AoArch: Aortic arch
Art trunk: Arterial trunk
AurTempA: Auriculo-temporal artery
AxA: Axillary artery
BA: Basilar artery
BrachA: Brachial artery
CarDuct: Carotid duct
CarTympA: Carotico-tympanic artery
Caud: Caudal
CCA: Common carotid artery
ChTN: Chorda tympani nerve
Contralat: Contralateral artery
ComNasCilA: Common nasal ciliary artery
ComTempCilA: Common temporal ciliary artery
Cran: Cranial
DA: Dorsal aorta
DieA: Diencephalic artery
DOA: Dorsal ophthalmic artery
Div: Division
DuctArt: *Ductus arteriosus*
ECA: External carotid artery
ExtMaxA: External maxillary artery
FrontA: Frontal artery
FrontN: Frontal nerve
HeubA: Recurrent artery of Heubner
HA: Hypoglossal artery
HyA: Hyoid artery
HyalA (CRA): Hyaloid artery (central retinal artery)
ICA: Internal carotid artery
InfAlvA: Inferior alveolar artery
Infraorb: Infraorbital
InnA: Innominate artery (brachio-cephalic trunk)
Interseg CervA: Intersegmental cervical artery
IntMammAIntAudA: Internal mammillary auditive artery
IntMaxA: Internal maxillary artery
LingA: Lingual artery
LNA: Longitudinal neural artery
MandA: Mandibular artery
MaxA: Maxillary artery
MCA: Middle cerebral artery
MedStrA: Medial striate artery
MesA: Mesencephalic artery
MetA: Metencephalic artery
MMA: Middle meningeal artery
N: Nerve
OA: Ophthalmic artery
OccA: Occipital artery
Olf: Olfactory
OlfA: Olfactory artery
Opt: Optic
OpV: Optic vesicle
OtA: Otic artery
OtV: Otic vesicle
P: Primitive
p1stCerv: First cervical segmental artery
PChA: Posterior choroidal artery
PCoA: Posterior communicating artery
Plac: Placode
PICA: Postero-inferior cerebellar artery
Pin: Pineal primordium
PostAurA: Posterior auricular artery
PulmA: Pulmonary artery
Reg: Regressing
Pulm trunk: Pulmonary trunk
Remn: Remnant
SA: Stapedial artery
SA (max): Mandibulo-maxillary division of stapedial artery
SCA: Superior cerebellar artery
SHA: Superior hypophyseal artery
SubclA: Subclavian artery
SupPetN: Superior petrosal nerve
SupraorbA: Supraorbital artery
SupThyrA: Superior thyroid artery
TA: Trigeminal artery
ThyrA: Thyroid artery
TympN: Tympanic nerve
ThyrCervTr: Thyro-cervical trunk
VA: Vertebral artery
VidA: Vidian artery
VOA: Ventral ophthalmic artery
VPA: Ventral pharyngeal artery
